# Computational investigation of unsaturated ketone derivatives as MAO-B inhibitors by using QSAR, ADME/Tox, molecular docking, and molecular dynamics simulations

**DOI:** 10.55730/1300-0527.3360

**Published:** 2021-12-18

**Authors:** Abdellah EL AISSOUQ, Mohammed BOUACHRINE, Abdelkrim OUAMMOU, Fouad KHALIL

**Affiliations:** 1 LPME Laboratory, Faculty of Science and Technology, Sidi Mohamed Ben Abdellah University, Fez, Morocco; 2 LIMOME Laboratory, Faculty of Sciences Dhar El Mahraz, Sidi Mohamed Ben Abdellah University, Fez, Morocco; 3 MCNS Laboratory, Faculty of Sciences, Moulay Ismail University, Meknes, Morocco; 4 EST Khenifra, Sultan Moulay Sliman University, Morocco

**Keywords:** MAO-B inhibitors, Parkinson’s disease, ligand-based drug design, structure-based drug design

## Abstract

Unsaturated ketone derivatives are known as monoamine oxidase B (MAO-B) inhibitors, a potential drug target for Parkinson’s disease. Here, molecular modeling studies, including 2D-QSAR, ADMET prediction, molecular docking, and MD simulation, were performed on a new series of MAO-B inhibitors. The objective is to identify new MAO-B inhibitors with high inhibitory efficacy. The developed 2D-QSAR model was based on the descriptors of MOE software. The most appropriate model, using the partial least squares regression (PLS regression) method, yielded 0.88 for the determination coefficient (r^2^), 0.28 for the root-mean-square error (RMSE), and 0.2 for the mean absolute error (MAE). The predictive capacity of the generated model was evaluated by internal and external validations, which gave the Q^2^ and R^2^_test_ values of 0.81 and 0.71, respectively. The ability of a compound to be orally active was determined using the drug-likeness and ADMET prediction. The results indicate that most of the compounds have moderate pharmacokinetic characteristics without any side effects. Furthermore, the affinity of the ligands (unsaturated ketone derivatives) to the MAO-B receptor was determined using molecular docking. The top conformers were then subjected to MD simulation. This research may pave the way for the development of novel unsaturated ketone derivatives capable of inhibiting the MAO-B enzyme.

## 1. Introduction

Monoamine oxidase (MAO) is a flavin adenine dinucleotide (FAD)-dependent enzyme involved in the oxidation of monoamine neurotransmitters such as catecholamines (i.e. epinephrine, norepinephrine, and dopamine) and 5-hydroxy-tryptamine (i.e. serotonin, to their corresponding aldehydes with the formation of hydrogen peroxide (H_2_O_2_) [1] (ure 1). In mammals, this enzyme exists in two isoforms, MAO-A and MAO-B, encoded by two genes located on the X chromosome [2]. Human isoenzymes MAO-A (527 aa) and MAO-B (520 aa) display a high degree of amino acid sequence homology (share 70% amino acid identity) [3]. Each isoenzyme is the target of affective and neurogenerative disorders [4,5]. MAO-A inhibitors are used to treat behavioral disorders, particularly depression [6,7]. In contrast, MAO-B inhibitors such as selegiline and rasagiline are used in the treatment of Parkinson’s disease (PD). MAO-B inhibitors are also involved in the treatment of Alzheimer’s disease (AD) [8,9] and other neurodegenerative disorders [10]. However, MAO-B drugs are irreversible and cause pharmacological side effects in the long-term treatment of PD [11]. Therefore, the development of reversible MAO-B inhibitors, such as the recent anti-Parkinson drug safinamide [12], is required.

**Figure 2:**
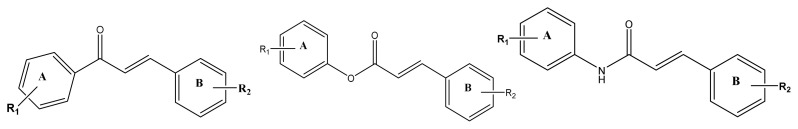
Chemical structures of chalcones (1), α, β-unsaturated ester derivatives (2), and α, β-unsaturated amide derivatives (3).

To search for new MAO-B inhibitors, chalcone scaffolds are selected. Chalcones are a class of polyphenolic derivatives belonging to the flavonoid family, their structure consisting of two aromatic cycles linked by a three-carbon α, β-unsaturated carbonyl system (Figure 2). They exhibit a wide range of pharmacological activities, including anticancer [13], antiinflammatory [14], antidiabetic [15], antioxidants [16, 17], antimalarial [18], anti-HIV [19] and anti-MAO-B [20].

Computer-aided drug design (CADD) approaches have recently become essential in drug discovery. These techniques proved to be effective in various stages of the drug development process, reducing both the cost and time required to develop a drug compared to conventional methods [21]. In general, CADD approaches are classified into two types: structure-based drug design (SBDD) and ligand-based drug design (LBDD). When the 3D structure of the protein is available, SBDD methods such as homology modeling, molecular docking, and molecular dynamics simulation are used [22]. On the other hand, LBDD methods such as quantitative structure-activity relationship (QSAR), similarity search, and pharmacophore modeling are applied when the 3D structure of the protein is not available. With the help of these methods, several approved drugs have been developed [23–28]. For example, the discovery of amprenavir as a potential inhibitor of the human immunodeficiency virus (HIV) protease using protein modeling and MD simulations [29], imatinib as an inhibitor of the tyrosine kinase using SBDD [30], amprenavir as a potential inhibitor of the human immunodeficiency virus (HIV) protease using protein modeling and MD simulations [31,32], thymidylate synthase inhibitor, raltitrexed against HIV using the SBDD approach [33], Norfloxacin as an inhibitor of topoisomerase II, IV using QSAR modeling [32], and darolutamide as an inhibitor of androgen receptor using docking and MD simulations [34].

In this work, a new series of chalcones and their derivatives were synthesized by Choi and coworkers [35]. The synthesized compounds are known as monoamine oxidase inhibitors (MAOI). They have a wide range of activity (0.016 < IC_50_ (μM) < 8.39). To understand the relationship between the chemical structures and their related activities, 2D-QSAR was performed, based on the descriptors of molecular operating environment (MOE) software. The pharmacokinetic properties of compounds were analyzed using the drug-likeness and the ADMET (adsorption, distribution, metabolism, excretion, and toxicity) prediction. Docking study was also displayed to find out the binding modes of the unsaturated ketone derivatives in the active site of monoamine oxidase-B (MAO-B) receptor. Finally, the dynamic behavior and stability of ligand-receptor complexes were evaluated using molecular dynamics (MD) simulation. In this study, the combination of ligand and structure-based drug design may be helpful to develop and design new unsaturated ketone candidates as MAO-B inhibitors.

## 2. Materials and methods

### 2.1. Dataset preparation and structure optimization

**Figure 3:**
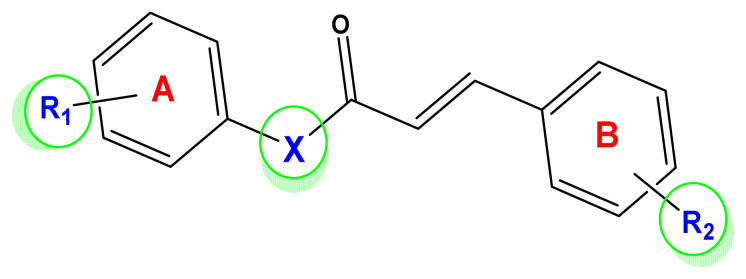
Core substructure of unsaturated ketone derivatives.

A dataset of unsaturated ketone derivatives synthetized by Choi and coworkers was selected [35]. The IC_50_ values were converted into corresponding pIC_50_ values (pIC_50_ = log (1/IC_50_.) and then used as a dependent variable in this study. The selected compounds are known as monoamine oxidase inhibitors and display a wide range of activity (3 log units). The dataset was randomly split into a training set (80% of the dataset) for 2D-QSAR model generation and a test set (20% of the dataset) for testing the predictive ability of the generated model. The core substructure of these compounds is shown in Figure 3.

Before modeling, the 3D chemical structures were designed using Marvin Sketch software. Energy minimization and calculations were performed using SYBYL-X 2.1 software [36]. All compounds were energy minimized using the conjugate gradient procedure based on the tripos force field with a convergence criterion value set to 0.01 kcal/mol Å [37]. The maximum number of iterations was set to 2000. Partial atomic charges were added, using Gasteiger-Hücke charges [38]. The chemical structures and their corresponding activity are presented in Table 1.

### 2.2. 2D-QSAR analysis

#### 2.2.1. Molecular descriptors calculation

The 2D-QSAR model was constructed using MOE descriptors. For each compound, a total of 354 descriptors were calculated. Then, the descriptors with a zero or equal variance predictor and the descriptors with a constant value for all observations were deleted. The number of descriptors was reduced by removing descriptors with a low correlation with the pIC_50_ value. The multicollinearity of the selected descriptors was verified by calculating the variation inflation factors (VIF), which can be calculated as follows:


Eq. 1 $$VIF=\frac{1}{1-r^{2}}$$

Where r^2^ is the multiple correlation coefficient of one descriptor’s effect regressed on the remaining molecular descriptors. If the VIF value is greater than 5 (VIF > 5), multicollinearity is very high [39].

#### 2.2.2. Model generation

After selecting the most appropriate descriptors, PLS method was used to build the linear 2D-QSAR model. The MOE descriptors were used as independent variables (X variables), while MAO-B inhibitory activity (pIC_50_) was employed as the dependent variable (Y variables). Both variables are related by the following equation below:


Eq. 2 $$Y=a_{0}+a_{1}X_{1}+a_{2}X_{2}+\cdots +a_{n}X_{n}$$

The developed QSAR model is evaluated by the squared correlation coefficient (r^2^), the adjusted squared correlation coefficient (r^2^_a_), the root-mean-square error (RMSE), the mean absolute error and the fisher value (F).[Fig f1-turkjchem-46-3-687]


Eq. 3 $$r^{2}=1-\left[\frac{\sum_{i}{\left(Y_{i}\;\text{obs}-Y_{i}\text{pred}\right)}^{2}}{\sum_{i}{\left(Y_{i}\;\text{obs}-{\bar{\text{Y}}}_{\text{i}}\;mean\right)}^{2}}\right]=1-\frac{SSE}{SST}$$

Eq.4 $$r_{a}^{2}=\frac{(n-1)\times r^{2}-p}{n-1-p}$$

Eq.5 $$RMSE=\sqrt{\frac{\sum_{I=1}^{n}\left(\text{Yi obs}-\text{Yi pred}\right)}{n}}$$

Eq.6 $$MAE=\frac{1}{n}\sum_{I=1}^{n}|\text{Yi obs}-\text{Yi pred}|$$

Eq.7 $$F=\frac{\frac{\sum_{i}{\left(Y_{i}\text{ pred}-{\bar{\text{Y}}}_{\text{i}}\;mean\right)}^{2}}{p}}{\frac{\sum_{i}{\left(Y_{i}\;\text{obs}-\text{Yi pred}\right)}^{2}}{N-P-1}}$$

In the above equations, Yi obs and Yi pred are the observed and predicted activities (pIC_50_ obs and pIC_50_ pred) for i^th^ compound in the training set, Ȳ_i_ mean is the average activity ( $\bar{{\text{pIC}}_{5}}$ mean) of the compounds in the training set, SSE is the residual of squares, SST is the total sum of squares, n is the total number of compounds in the training set, and p is the number of descriptors in the generated model. For a good model, the value of r^2^ should be closed to 1, the values of RMSE and MAE should be closed to 0, and the value of F should be high. The generated model is used to understand how the activity changes when any one of the descriptors is varied.

### 2.3. QSAR Model validation

#### 2.3.1. Internal validation

##### Leave-one-out cross validation

In order to judge the quality and goodness of the generated QSAR model, the leave-one-out (LOO) cross validation process is performed [40, 41]. In this process, one compound is primarily eliminated from the training set. Then, the QSAR model is built based on the remaining compounds (n-1), and the activity of the deleted compound is predicted by the established QSAR model. This process is repeated until all the compounds have been removed once. The performance of the QSAR model is measured by the cross validated correlation coefficient (Q^2^_LOO_), which is calculated by the following equation below [42]:


Eq.8 $${\text{Q}}_{\text{LOO}}^{2}=1-\left[\frac{\sum_{i}{\left(Y_{i}\;\text{obs}-Y_{i}\text{pred}\right)}^{2}}{\sum_{i}{\left(Y_{i}\;\text{obs}-{\bar{\text{Y}}}_{\text{i}}\;mean\right)}^{2}}\right]$$

In the above equation, Yi obs and Yi pred are the observed and predicted activities (pIC_50_ obs and pIC_50_ pred) for i^th^ compound in the training set, based on the LOO cross validation method, Ȳ_i_ mean is the average activity ( $\bar{{\text{pIC}}_{5}}$) of the compounds in the training set. The value of Q^2^_LOO_ reflects the quality of the model and it should be >0.5 (Q^2^_LOO_ > 0.5) [43].

##### R^2^_m (LOO)_ parameter

A high value of Q^2^_LOO_ does not indicate that the observed and predicted activities are close to each other. To solve this problem, and to better indicate the internal validation of the QSAR model, R^2^_m (LOO)_ and $R_{m\;(LOO)}^{\prime 2}$ metrics are calculated [44].


Eq.9 $${{\text{R}}_{\text{m}}}^{2}=R^{2}\times (1-\sqrt{\left(R^{2}-R_{0}^{2}\right)}$$

Eq.10 $${{R^{\prime }}_{\text{m}}}^{2}=R^{2}\times (1-\sqrt{\left(R^{2}-R_{0}^{\prime^{2}}\right)}$$

Where, R^2^ and R_0_^2^ are the squared correlation coefficient values between the observed and calculated (leave-one-out) activities with and without intercept, respectively. The parameter ${\text{R}}_{0}^{\prime^{2}}$ has the same meaning as R_0_ but with the axes reversed. The value of R^2^_m (LOO)_ and $R_{m\;(LOO)}^{\prime^{2}}$ should be more than 0.5.

#### 2.3.2. Y-randomization test

To ensure the robustness of the developed QSAR model, a Y-randomization test is performed [45]. The values of pIC_50_ are randomly permuted and a new QSAR model is generated using the original descriptors [46]. The new QSAR models are expected to have significant low R^2^ and Q^2^ values for several trials, which confirm that the generated QSAR model is robust and not due to a chance correlation. Another parameter, ^c^R_p_^2^ is also calculated by the following equation below:

Eq.9 Rcp2=R*R2-(Average Rr)2

Where, R_r_ is the average ‘R’ of random models. The ^c^R_p_^2^ value should be more than 0.5 to pass this test.

#### 2.3.3. External validation

##### Test set (r^2^_test_)

The predictability of the elaborated QSAR model is evaluated by the external validation or test set. This process consists in keeping a set of compounds not included in the model generation and their activity values are predicted by the generated QSAR model. The performance of the external validation is evaluated by the squared correlation coefficient of prediction (R^2^_test_), which is calculated by the following equation [47].


Eq.10 $${\text{r}}_{\text{test}}^{2}=1-\left[\frac{\sum_{i}{\left(Y_{i}\;\text{obs }(\text{test})-Y_{i}\text{pred }(\text{test})\right)}^{2}}{\sum_{i}(Y_{i}\;\text{obs }(\text{test})-{\bar{\text{Y}}}_{\text{i}}\;mean\;(train)){\;}^{2}}\right]$$

Where, Y_i obs (test)_ and Y_i pred (test)_ represent the observed and predicted activity (pIC_50 obs_ (test) and pIC_50 pred_ (test)) of the i^th^ compound in the test set, respectively and Ȳ_i_ mean ( $\bar{pI}C50\;mean$) represents the mean activity of the compounds in the training set. The value of the R^2^_test_ should be more than 0.5, for a power predictability.

##### Golbraikh and Tropsh’s criteria

The external predictability of the generated QSAR model is also evaluated by several parameters. According to Golbraikh and Tropsha [48], a QSAR model is considered satisfactory if all the following conditions are satisfied:

- Q^2^ > 0.5
- R^2^_test_ > 0.6
-
$\frac{r^{2}-r_{0}^{2}}{r^{2}}<0.1$ and 0.85 < k < 1.15 or $\frac{r^{2}-{{r_{0}}^{\prime }}^{2}}{r^{2}}<0.1$ and 0.85 < *k*′ < 1.15
- |*r*^2^ − *r*_0_^′2^| < 0.3

Where k is the slop of the plot of the observed and predicted values of compounds via the origin and *k*′ is the reversed axes intercept.

#### 2.3.4. R^2^_m_ (test) parameter

A high value of R^2^_test_ does not indicate that the observed and predicted activities are close to each other. To solve this problem, and to better indicate the external validation of the QSAR model, R^2^_m (test)_ and $R_{m}^{\prime^{2}}\;(\text{test})$ metrics, similar to R^2^_m (LOO)_ and $R_{m\;(LOO)}^{\prime^{2}}$ are calculated [44]. For an acceptable prediction, the value of $\Delta R_{m}^{2}\;(\text{test})$ should be lower than 0.2, provided that the value of $\bar{R_{m}^{2}}$ is more than 0.5 [44].


Eq.11 $$\bar{R_{m}^{2}}=\frac{\left(R_{m}^{2}+R_{m}^{\prime 2}\right)}{2}$$

Eq.12 $$\Delta R_{m}=|R_{m}^{2}-R_{m}^{\prime 2}|$$

#### 2.3.5. Q^2^ (F2) metric

The Q^2^ (F2) metric can be calculated by the following equation below [49]:


Eq.13 $${\text{Q}}_{\left(\text{F}2\right)}^{2}=1-\frac{\sum_{i}{\left(Y_{i}\;\text{obs }(\text{test})-Y_{i}\text{pred }(\text{test})\right)}^{2}}{\sum_{i}{\left(Y_{i}\;\text{obs }(train)-{\bar{\text{Y}}}_{\text{i}}test\right)}^{2}}$$

Where, Ȳ_i_ represents the mean observed data of the test set compounds. The value of Q^2^ (F2) should be more than 0.5.

### 2.4. Applicability domain

The applicability domain is defined as a theoretical region in chemical space, including both model variables and modeled response [50,51]. The applicability domain is based on the calculation of the leverage value (h_i_) of each compound i, for which a QSAR model is used to predict its activity:


Eq.14 $${\text{h}}_{\text{i}}={{\text{x}}_{\text{i}}}^{\text{T}}{\left({\text{X}}^{\text{T}}\text{X}\right)}^{-1}{\text{x}}_{\text{i}}\;\;\;(\text{i}=1,\ldots ,\text{n}),$$

In Eq.14, x_i_ is the descriptor row-vector of a query compound, and X is the n*(k-1) matrix of k descriptor values for n data set compounds. A compound i was considered outside the applicability domain when the leverage value (h_i_) of this compound is greater than the critical value (h*) (h* = 3(d+1)/n, where d is the number of variables and n is the number of compounds in the training set). Conversely, a compound i is considered inside the applicability domain when h_i_ is lower than the h*.

### 2.5. Drug likeness and ADMET analysis

Drug likeness and ADMET (absorption, distribution, metabolism, excretion, and toxicity) analysis were performed using pkCSM [52] and SWISSadmet [53] web servers. The drug likeness of all compounds was verified using the Lipinski’s rule of five [54], veber [55], Egan, Muegge [55], and Ghose [56] rules. The pharmacokinetic properties such as blood-brain barrier (BBB) permeability, human intestinal absorption (HIA), water solubility (log mol/L), CYP450 substrate and its inhibitor (CYP2D6, CYP3A4, CYP1A2, CYP2C19 and CYP3A4), CaCo-2 permeability and toxicity were evaluated using ADMET analysis. Some other physicochemical properties such as molecular weight (MW), octanol-water partition (LogP), number of hydrogen bond acceptors (HBA), number of hydrogen bond donors (HBD), and topological polar surface area were also verified.

### 2.6. Docking study

Molecular docking is one of the most virtual screening methods, especially when the 3D structure of the receptor is available [57–59]. Here, the X-ray diffraction structure of monoamine oxidase B (MAO-B) was downloaded from the protein database bank (PDB ID: 2BK3) [60]. All preparations were carried out using the AutoDock tool [61]. The ligand was extracted from the protein and the water molecules were removed. Gast-Huck charges and polar hydrogen were added to the crystal structure. Then, a grid box centered on the catalytic site of the MAO-B receptor was created with a dimension of 40 × 40 × 40 Å in x, y, and z directions, respectively. Finally, the optimized ligands were converted to the pdbqt format and docked into the binding site of MAO-B using AutoDock vina [62]. The results were analyzed and visualized using PyMol and Discovery Studio 2017 R2 software’s.

### 2.7. Molecular dynamics simulations

The molecular dynamics simulations were performed using GROMACS 5.1.4 package [63]. The topology files of the ligand and the protein were generated using the CHARMM General Force Field (CGenFF) server and the ‘pdb2gmx’ script, respectively [64]. The simulations were run using the CHARMM36 all-atom (March, 2019) force field [65] in a triclinic box with a distance of 1.0 nm and a TIP3P water model solvated system [66]. The neutralization of the system was performed by adding sodium (Na+) or Chlorure (Cl^−^) ions. The energy minimization system was subjected to 50,000 steps using the steepest descent algorithm. Then, the production MD simulations were run for 20 ns for each simulation at a temperature of 300 k, a pressure of 1 bar and a time step of 2 fs.

## 3. Results and discussion

### 3.1. 2D-QSAR analysis

#### 3.1.1. Model generation

2D-QSAR model was built based on the training set compounds. The PLS method was used to generate the 2D-QSAR model by establishing a linear correlation between the most relevant descriptors (BCUT_SMR_2, LogP (o/w), SlogP_VSA4, and vsurf_IW3) and the inhibitory activity of MAO-B enzyme (pIC50). The linear equation connecting the selected descriptors to MAO-B inhibitory activity is presented below.


Eq. 15 $$\begin{array}{l} \text{pIC}50=-3,754+8,469*\text{BCUT}\_\text{SMR}\_2+0,636*\text{logP}\left(\frac{\text{o}}{\text{w}}\right)+0.057*\text{SlogP}\_\text{VSA}4-0,442*\text{vsurf}\_\text{IW}3\\ \text{N}=20,\;\text{RMSE}=0.28,\;{\text{r}}^{2}=0.88,\;{{\text{r}}_{\text{Adjusted}}}^{2}=0.84,\;\text{F model}=27.76,\;\text{p}<0.0001,\;\alpha =5\%,\;{{\text{r}}^{2}}_{\text{cv}}=0.80,\;{\text{RMSE}}_{\text{cv}}=0.31,\;{{\text{r}}^{2}}_{\text{test}}=0.71 \end{array}$$

In the above equation, N is the number of compounds in the training set, RMSE is the root mean square error, and F is the Fisher value.

The best generated model explains 88% (r^2^ = 0.88) of the total variance in the training set with small values of RMSE and MAE (RMSE = 0.28 and MAE = 0.2). The high value of the F-test (F = 27.76) indicates that the generated model is statistically significant. Also, the significance of each descriptor in Eq.15 was verified by calculating the value of VIF and p (Table 2). All descriptors in the generated model have acceptable values of VIF and p (VIF < 5 and p < 0.05), indicating a good significant relationship between the modeled response and the selected descriptors. The correlation matrix of the selected descriptors is shown in Table 3. Table 4 shows the values of selected descriptors, experimental activity, and predicted activity.

#### 3.1.2. 2D-QSAR model validation

**Figure 4:**
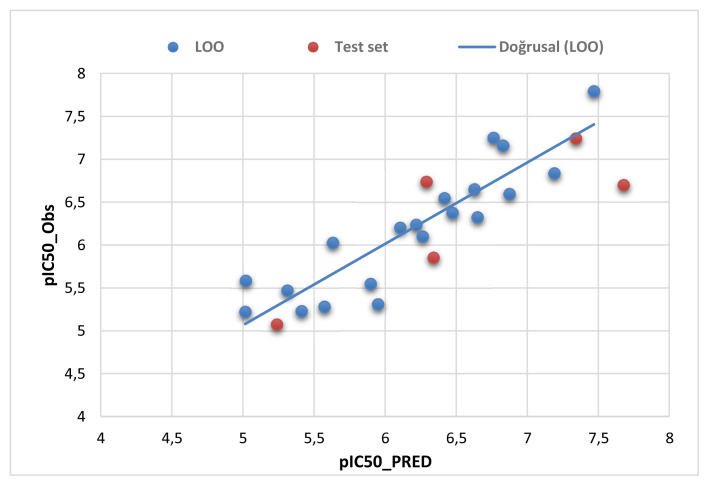
Plot of predicted and observed activity. The blue dots denote internal validation (LOO), whereas the orange dots represent external validation.

The predictability of the generated model was verified by internal and external validations. The statistical parameters are listed in Table 5. The performance of internal validation was determined by leave-one out cross validation. This approach was used to predict the activity (pIC_50_LOO_) of each compound in the data set using the model established by (n-1) compounds. Our developed model is predictive, as evidenced by the results (Q^2^_LOO_ = 0.81, RMSE _LOO_ = 0.31 and MAE_LOO_ = 0.27). The quality of Q^2^_LOO_ was also verified by calculating the R^2^_m_ (R^2^_m_ = 0.57 > 0.5) parameter. To ensure the robustness of the developed model, a y-randomization test was applied. Several random shuffles of pIC_50_ were performed. The results are summarized in Table S1 (see supplementary data). The low average R^2^ and Q^2^ (R^2^ = 0.15 and Q^2^ = −0.52) and the high value of ^c^R_p_^2^ (^c^R_p_^2^ = 0.81 > 0.5), indicate that the good results in our original model are not due to a chance correlation. The external validation of the developed model was verified by a test set of 5 compounds. The results (r^2^_test_ = 0.71) indicate that the developed model is capable of predicting the activity of new untested compounds. Also, the goodness of external validation was verified by calculating the Q^2^_(F2)_ parameter, which gave a value greater than 0.5 (Q^2^_(F2)_ = 0.52). The plot of the experimental versus the predicted activities for the internal and the external validations is shown in Figure 4.

#### 3.1.3. 2D-QSAR descriptors interpretation

**Figure 5:**
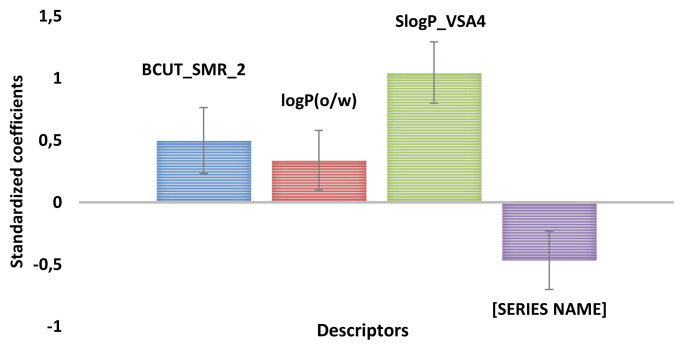
Contribution of descriptors in the generated 2D-QSAR model.

In order to understand the relationship between BCUT_SMR_2, LogP (o/w), SlogP_VSA4, and vsurf_IW3 descriptors and the inhibitory activity of the MAO-B enzyme, it is necessary to explain the meaning of each descriptor in Eq. 15. Figure 5, shows the contribution of each descriptor in Eq. 15.

As described in Figure 5, the most important descriptor in the established 2D-QSAR model is SlogP_VSA4. This descriptor represents the sum of approximate accessible van der Waal’s surface area i such that logP for atom i is in the range (0.1 to 0.15). The positive contribution (+ 0.057) and high correlation (r = 0.72) with the inhibitory activity, indicate that compounds with high accessible van der Waals surface area could increase the inhibitory activity of the MAO-B enzyme. A deep analysis of chemical structures and their experimental activity indicate that the SlogP_VSA4 descriptor is related to the number of fluorine (F) in R1 and R2 positions. From the experimental dataset (Table 1), compounds with R1 = F (16a, 16b, 16c, 17a, and 18a) have the same value of SlogP_VSA4 (SlogP_VSA4 = 6.3716) and compounds with R2 = CF3 have the same value of SlogP_VSA4 (SlogP_VSA4 = 33.4189). To ensure that the SlogP_VSA4 descriptor is related to the number of fluorine, we compared compounds 10b and 11b, which have the same substituent in the R2 position (R2 = CF3) but differ in the R1 position (10b: R1 = −OCH3, 11b: R1 = OH). The value of the SlogP_VSA4 descriptor is the same in both compounds (SlogP_VSA4 = 33.4189).

The second most important descriptor in the generated 2D-QSAR model is BCUT_SMR_1. The BCUT descriptor using atomic contribution to molar refractivity (using the Wildman and Crippen SMR method) instead of partial charge [67]. The positive influence (+ 8.469) of this descriptor in Eq. 15, reflects the importance of atomic properties that govern the intermolecular interactions (atomic charge, atomic polarizability, and atomic hydrogen bonding ability) on the inhibitory activity of MAO-B enzyme [68].

The next descriptor in the generated 2D-QSAR model is Vsurf_IW3, which describes the hydrophilic integy moment, calculated at −0.6 kcal/mol [69]. The integy moment of this descriptor measures the unbalance between the center of mass of a compound and the barycenter of specific regions of the surface [70]. The negative contribution (−0.442) of this descriptor in Eq. 15, indicates that the integy moment of compounds should be smaller. To reduce the integy moment, the polar moieties should be close to the center of mass or at the opposite ends of the compound. This may explain the significant increase in activity of compounds 10d (pIC_50_ = 6.701, ortho-R2 = CF3), 10c (pIC_50_ = 6.8386, meta-R2 = CF3), and 10b (pIC_50_ =7.7959, para-R2 = CF3). Moreover, compounds with only one hydrated region (12a, 12b and 19a), show high values of Vsurf_IW3 and consequently, low activity.

The LogP (o/w) (log of the octanol/water partition coefficient) is a descriptor for measuring the overall hydrophobicity of compounds [71]. The positive contribution (+ 0.636) of this descriptor in Eq. 15, indicates that a decrease in the lipophilicity of compounds could increase the inhibitory activity of the MAO-B enzyme.

#### 3.1.4. Applicability domain

**Figure 6:**
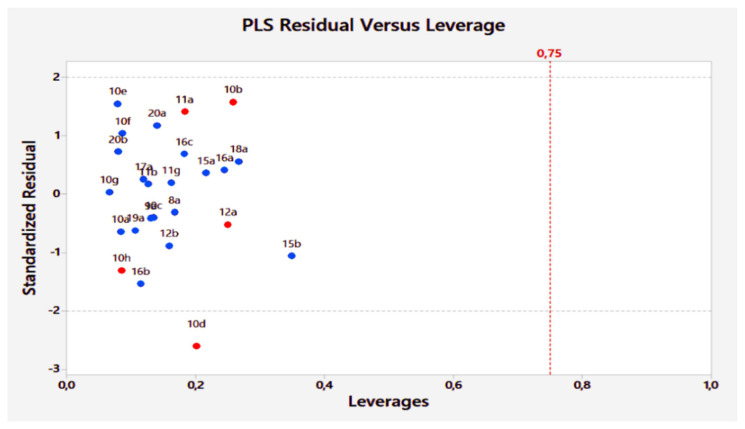
Applicability domain of the generated 2D-QSAR model. The blue dots represent the training set compounds and the red dots represent the test set compounds.

The applicability domain of the developed 2D-QSAR model was generated, using William’s plot (Figure 6), taken by MINITAB. 17 software [72]. As described in this figure, all compounds are in the applicability domain, except compound 10d, which has a standardized residual value greater than ±2*σ*. Although this compound is considered outside the applicability domain.

### 3.2. Physicochemical properties and drug-likeness

Physicochemical properties and drug-likeness results are summarized in Table S2 (see supplementary data). All compounds show MW values less than 500 Da (224.25 < MW < 309.26), LogP values less than 5 (3.28 < LogP < 4.61), HBA less than 10 (2 < HBA < 5), HBD less than 5 (0 < HBD < 2), and TPSA less than 140 Å (26.30 Å < TPSA < 49.33 Å). This result revealed that these compounds are very likely to be orally active. Also, the results of drug likeness showed that all compounds respect the Lipinski rule of five, Veber, Egan, and Muegge rules without any violations. However, compounds 8b, 9b, 10b, 10c, 16a, 16b, 16c, 17a, and 18a showed one violation for the Ghose rule (WLOGP>5.6).

### 3.3. ADMET prediction

The pharmacokinetic (PK) properties and toxicity were envaulted using ADMET analysis. The results are summarized in Table S3 (see supplementary data). The moderate values of LogBB reveal that these compounds may be effective for treating the neurodegenerative disease. The values of human intestinal absorption (HIA) (HIA > 30%) indicate that these compounds are highly absorbed. Also, the absorption rate of the studied compounds was evaluated by the Caco-2 cell permeability parameter. The results indicate that all compounds show high Caco-2 cell permeability (Caco-2 > 0.90). In addition, the metabolism of the studied compounds was verified by the inhibitory or substrate behavior of the cytochrome P450 enzymes (CYPs). This enzyme plays a major role in the oxidation process and facilitates the excretion of foreign organic compounds, including drugs. All the compounds were found to be a substrate of 3A4, while no compounds were found to be a substrate of 2D6. Moreover, results of inhibition studies indicate that no compounds were found to inhibit 2D6, whereas, all of them were found to inhibit 1A2, 2C19, and 2C9, except compounds 11a, 11g, and 19a. The toxicity analysis, including AMES toxicity and hepatoxicity indicates that the predicted compounds are not harmful. However, the maximum tolerated dose in human was in the range of 0.26–1.23 mg/kg/day.

### 3.4. Molecular docking analysis

#### 3.4.1. Docking validation

In order to validate the binding site of the crystal structure, docking validation (or redocking) was applied. First, the native ligand was extracted from its PBD structure (PDB ID: 2BK3). The docking parameters were generated using AutoDock tools. Then, the native ligand was redocked into the same binding site of the crystal structure, using AutoDock 4.1. The best obtained pose gave the root mean square deviation (RMSD) value of 1.57, using 50 iterations. The superposition between the native and the redocked ligands is shown in Figure S1 (see supplementary data).

#### 3.4.2. Binding modes interactions and affinity of the studied compounds

**Figure 7:**
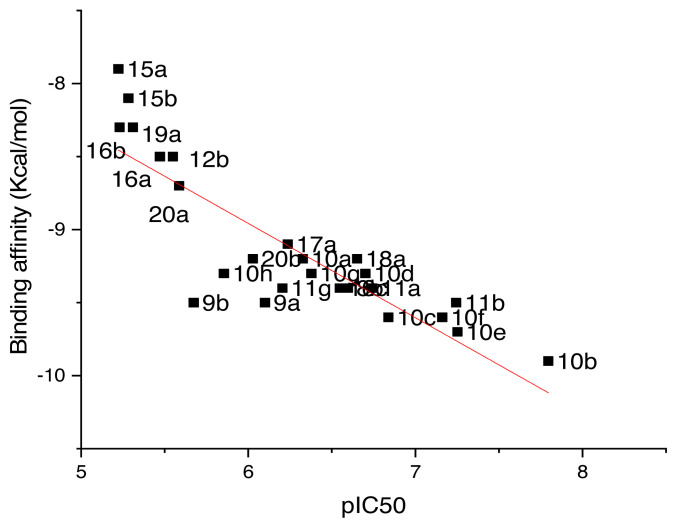
Correlation between the binding free energy values and the pIC50 values.

After docking validation, the dataset of compounds was used to generate the docking study. The results are summarized in Table S4 (see supplementary data). The values of binding free energy revealed that unsaturated ketone derivatives showed significant stability in the binding site of the MAO-B enzyme. In addition, the correlation between the binding free energy and the pIC_50_ values (r^2^ = 0.73) showed that the docking results are in agreement with the activity values. Figure 7, illustrates the correlation between the binding free energy and pIC_50_ values.

**Figure 8:**
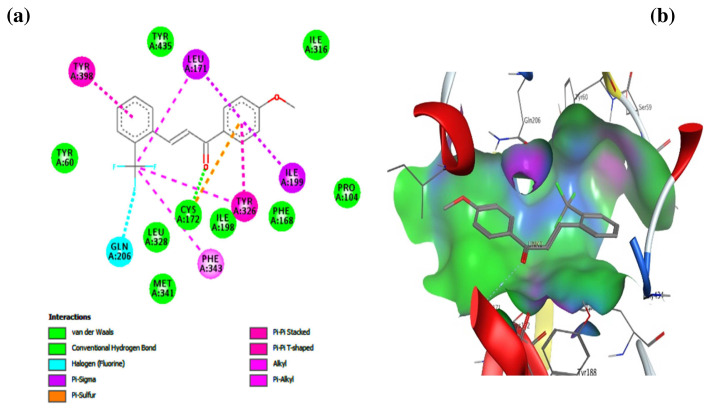
Docking analysis of compound 10b. (a) 2D view of binding site interactions, (b) 3D view of the binding conformation.

**Figure 9:**
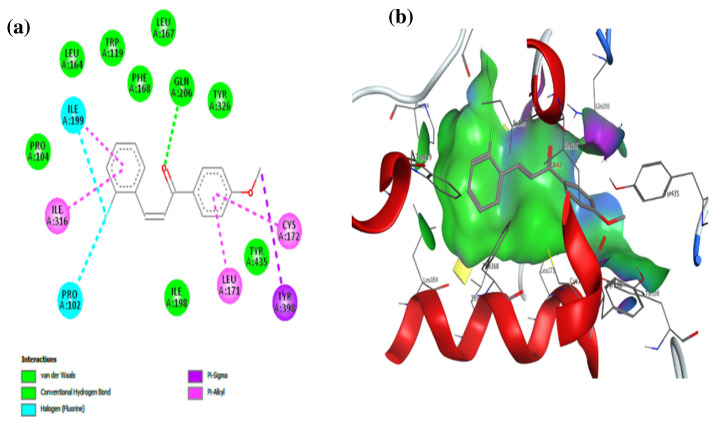
Docking analysis of compound 10e. (a) 2D view of binding site interactions, (b) 3D view of the binding conformation.

In order to predict whether and how unsaturated ketone derivatives bind to the MAO-B active site, compounds 10b and 10e (the most potent inhibitors in the dataset) were used. The results are shown in Figures 8 and 9. As described in Figure 8, compound 10b was fixed in the binding pocket of the MAO-B enzyme by several types of interactions. The most important are the conventional hydrogen bond interaction with CYS 172, the halogen interaction with GLY 204, and hydrophobic interactions (Pi-Pi stacked, Pi-Pi T-shaped, Alkyl, Pi-alkyl, and Pi-sigma) with TYR 398, PHE 343, TYR 326, CYS 172, ILE 199, and LEU 171. These types of interactions may confirm why this compound displays a low binding affinity (−9.9 Kcal/mol). The low binding affinity of compound 10e (−9.7 Kcal/mol) was also related to the type of interactions that it has with the MAO-B receptor. As described in Figure 9, compound 10e was fixed in the binding pocket of the MAO-B receptor with the same type of interactions (halogen interactions with the fluorine at the ortho-R1 position, conventional hydrogen bond interaction with the ketone group of α, β-unsaturated, and hydrophobic interactions at the R2 position) as that of compound 10b. The values of binding affinity of the studied compounds are related to the number of interactions, the types of interactions, and the distance between the ligand and a particular amino acid.

### 3.5. Molecular dynamics simulation

**Figure 10:**
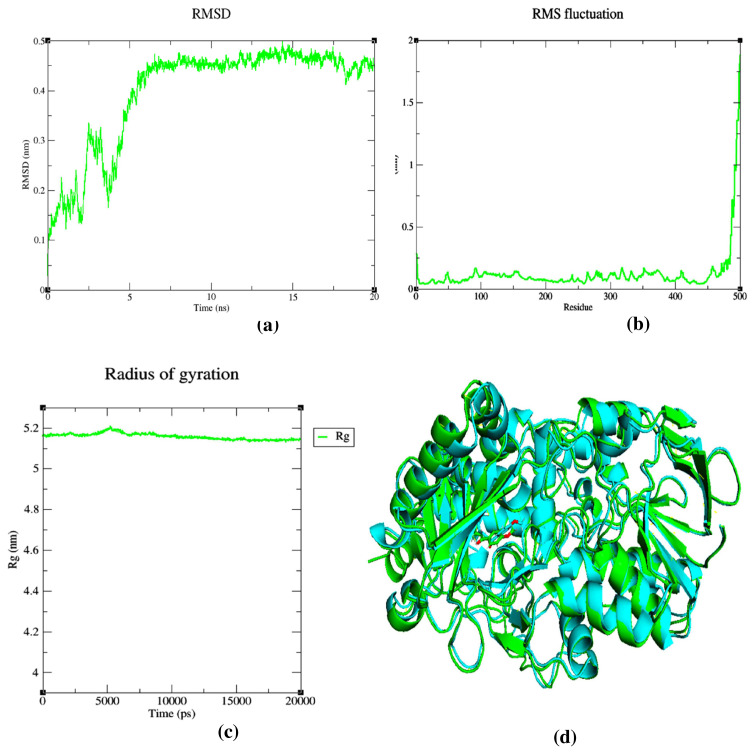
Molecular dynamics simulations of the 10b_2BK3 complex. (a) represents the RMSD plot, (b) represents the RMSF plot, (c) represents the Rg plot, and (d) represents the superposition of the final complex structure after MD simulation of 20 ns (green color) and initial complex structures before MD simulation (blue color).

In order to validate the results of molecular docking and to affirm the stability of docked compounds in the binding pocket of the MAO-B receptor, the most active compound in the dataset (compound 10b) was subjected to MD simulation. The system was employed for 20 ns time scale simulation. The results are shown in Figure 10. The plot of the root means square deviation (RMSD) (Figure 10a) indicates that the 10b_2BK3 complex attained stability at 7 ns. Then, it remained constant during the MD simulation. The average RMSD value is 0.26 nm with a maximum value of 0.49 nm and a minimum value of 0.37 nm. The root means square fluctuation (RMSF) of the 10b_2BK3 complex is also determined (Figure 10b**)**. This parameter is an indicator of residual flexibility. We conclude that all amino acid residues had RMSF values less than 0.1 nm, with the exception of residues 470–500, which had RMSF values greater than 0.1 nm. The stability of the system was also verified by plotting the radius of gyration (Rg) (Figure 10c). The Rg value of the complex is in the range of 5.15 – 5.2 nm suggesting that these complexes are stable and compact during the 20 ns of the MD simulation. In addition, and from Figure 10d, we can see that the main secondary structural elements of the ligand and the protein in the 10b_2BK3 complex remained close to its initial structure before the MD simulation.

## 4. Conclusion

In this paper, molecular modeling studies including 2D-QSAR, ADMET, molecular docking, and molecular dynamics simulation were applied on a series of unsaturated ketone derivatives as MAO-B inhibitors. The PLS technique was utilized to build the 2D QSAR model, which employed the pIC50 values as a dependent variable and the MOE descriptors as independent variables. The results revealed the importance of BCUT_SMR_2, logP (o/w), SlogP_VSA4 and vsurf_IW3 descriptors in describing the activity values. The pharmacokinetic characteristics of compounds were also studied using drug likeness and ADMET prediction. Molecular docking was carried out to investigate the binding site interactions between ligands and the MAO-B receptor. The activity data exhibited a greater correlation (r^2^ = 0.73) with the binding free energy values. Finally, the active compounds were evaluated using MD simulation. The findings of this work clearly demonstrate the importance of unsaturated ketone derivatives in inhibiting MAO-B enzymatic activity, and they may open the way for the development of other inhibitory derivatives as Parkinson’s disease possible treatments.

Figure S1 The superposition between the native (green color) and the redocked (red color) ligands into the binding site of MAO-B enzyme.

**Table S1:** Y-randomization parameters after several runs.

Model	R	R ^2^	Q ^2^	Model	R	R ^2^	Q ^2^
Original	0.94	0.88	0.81	Random 11	0.28	0.08	−0.65
Random 1	0.55	0.30	−0.20	Random 12	0.29	0.09	−0.59
Random 2	0.11	0.01	−0.91	Random 13	0.47	0.22	−0.41
Random 3	0.44	0.19	−0.51	Random 14	0.50	0.25	−0.40
Random 4	0.23	0.05	−0.74	Random 15	0.54	0.29	−0.11
Random 5	0.38	0.14	−0.54	Random 16	0.28	0.08	−0.62
Random 6	0.39	0.15	−0.54	Random 17	0.32	0.10	−0.48
Random 7	0.50	0.25	−0.49	Random 18	0.34	0.12	−0.51
Random 8	0.57	0.32	−0.34	Random 19	0.35	0.13	−0.48
Random 9	0.33	0.11	−0.73	Random 20	0.39	0.15	−0.63
Random 10	0.23	0.05	−0.65				

**Table S2:** Physicochemical properties and drug-likeness of dataset compounds.

No	Physicochemical properties					Druglikeness					
	MW	Log P	HBA	HBD	TPSA	Lipinski	Ghose	Veber	Egan	Muegge	Bioavailability Score
8b	306.28	4.61	5	0	26.30	Yes 0 violation	No 1 violation: WLOGP>5.6	Yes	Yes	Yes	0.55
9a	238.28	3.59	2	0	26.30	Yes 0 violation	yes	Yes	Yes	Yes	0.55
9b	306.28	4.61	5	0	26.30	Yes 0 violation	No 1 violation: WLOGP>5.6	Yes	Yes	Yes	0.55
10a	238.28	3.59	2	0	26.30	Yes 0 violation	yes	Yes	Yes	Yes	0.55
10b	306.28	4.61	2	0	26.30	Yes 0 violation	Yes	Yes	Yes	Yes	0.55
10c	306.28	4.61	5	0	26.30	Yes 0 violation	No 1 violation: WLOGP>5.6	Yes	Yes	Yes	0.55
10d	306.28	4.61	5	0	26.30	Yes 0 violation	No 1 violation: WLOGP>5.6	Yes	Yes	Yes	0.55
10e	256.27	3.73	3	0	26.30	Yes 0 violation	yes	Yes	Yes	Yes	0.55
10f	272.73	4.24	2	0	26.30	Yes 0 violation	yes	Yes	Yes	Yes	0.55
10g	268.31	3.59	3	0	35.53	Yes 0 violation	yes	Yes	Yes	Yes	0.55
10h	268.31	3.59	3	0	35.53	Yes 0 violation	yes	Yes	Yes	Yes	0.55
11a	224.25	3.28	2	1	37.30	Yes 0 violation	yes	Yes	Yes	Yes	0.55
11b	292.25	4.30	5	1	37.30	Yes 0 violation	yes	Yes	Yes	Yes	0.55
11g	254.28	3.29	3	1	46.33	Yes 0 violation	yes	Yes	Yes	Yes	0.55
12b	257.71	3.99	4	1	29.10	Yes 0 violation	yes	Yes	Yes	Yes	0.55
15a	307.27	4.06	5	2	49.33	Yes 0 violation	yes	Yes	Yes	Yes	0.55
15b	273.71	3.69	2	2	49.33	Yes 0 violation	yes	Yes	Yes	Yes	0.55
16a	309.26	4.49	5	1	29.10	Yes 0 violation	No 1 violation: WLOGP>5.6	Yes	Yes	Yes	0.55
16b	309.26	4.49	5	1	29.10	Yes 0 violation	No 1 violation: WLOGP>5.6	Yes	Yes	Yes	0.55
16c	309.26	4.49	5	1	29.10	Yes 0 violation	No 1 violation: WLOGP>5.6	Yes	Yes	Yes	0.55
17a	309.26	4.49	5	1	29.10	Yes 0 violation	No 1 violation: WLOGP>5.6	Yes	Yes	Yes	0.55
18a	309.26	4.49	5	1	29.10	Yes 0 violation	No 1 violation: WLOGP>5.6	Yes	Yes	Yes	0.55
19a	224.25	3.30	2	0	26.30	Yes 0 violation	yes	Yes	Yes	Yes	0.55
20a	254.28	3.31	3	0	35.53	Yes 0 violation	yes	Yes	Yes	Yes	0.55
20b	288.73	3.96	3	0	35.53	Yes 0 violation	yes	Yes	Yes	Yes	0.55

LogP = octanol–water partition coefficient, MW = molecular weight, HBA = number of hydrogen bond acceptor, TPSA = topological polar surface area, HBD = number of hydrogen bond donor.

**Table S3:** ADMET prediction of all the compounds in the dataset.

Compounds	Absorption			Distribution	Metabolism							Excretion	Toxicity		
	Water solubility	Caco2 permeability	Intestinal absorption human (HIA)	Blood brain barrier permeability		CYP						Total clearance	AMES toxicity	hepatoxicity	Max. tolerated dose (human)
					2D6	3A4	1A2	2C19	2C9	2D6	3A4				
					Substrate		Inhibitor								
	Numeric (log mol/L)	Numeric (log Papp in 10 ^−6^ cm/s)	Numeric (% absorbed)	Numeric (log BB)	Categorical (Yes/No)							Numeric (log ml/min/kg)	Categorical (Yes/No)	Categorical (Yes/No)	Numeric (log mg/kg/day)
8b	−5.905	1.46	93.70	0.33	No	Yes	Yes	Yes	Yes	No	No	−0.003	No	No	0.83
9a	−4.66	1.45	97.68	0.39	No	Yes	Yes	Yes	Yes	No	No	0.23	No	No	1.05
9b	−6.04	1.48	94.41	0.35	No	Yes	Yes	Yes	Yes	No	Yes	0.044	No	No	0.91
10a	−4.73	1.07	98.12	0.38	No	Yes	Yes	Yes	Yes	No	No	0.24	No	No	1.02
10b	−6.02	1.11	94.85	0.33	No	Yes	Yes	Yes	Yes	No	Yes	0.055	No	No	0.88
10c	−6.02	1.11	94.85	0.34	No	Yes	Yes	Yes	Yes	No	Yes	0.045	No	No	0.88
10d	−6.03	1.11	94.85	0.34	No	Yes	Yes	Yes	Yes	No	Yes	0.035	No	No	0.88
10e	−4.97	1.31	97.35	0.35	No	Yes	Yes	Yes	Yes	No	No	0.19	Yes	No	1.007
10f	−5.41	1.08	96.46	0.32	No	Yes	Yes	Yes	Yes	No	No	−0.007	No	No	0.99
10g	−4.93	1.29	98.10	−0.13	No	Yes	Yes	Yes	Yes	No	No	0.33	Yes	No	0.97
10h	−4.97	1.10	99.00	−0.19	No	Yes	Yes	Yes	Yes	No	No	0.26	No	No	1.05
11a	−3.65	1.63	94.05	0.16	No	Yes	No	Yes	No	No	No	0.15	No	No	0.63
11b	−4.95	1.66	90.77	0.18	No	Yes	Yes	Yes	Yes	No	No	−0.04	No	No	0.5
11g	−3.73	1.35	93.93	−0.08	No	Yes	Yes	Yes	No	No	No	0.23	No	No	0.56
12b	−5.23	1.51	91.54	0.27	No	Yes	Yes	Yes	Yes	No	No	0.045	No	Yes	0.69
15a	−4.71	1.39	89.13	−0.08	No	Yes	Yes	Yes	Yes	No	Yes	0.074	No	Yes	0.26
15b	−.3.99	1.34	90.25	−0.052	No	Yes	Yes	Yes	Yes	No	No	−0.081	No	No	0.35
16a	−5.69	1.48	91.57	0.27	No	Yes	Yes	Yes	Yes	No	Yes	−0.071	No	Yes	0.74
16b	−5.64	1.48	90.91	0.26	No	Yes	Yes	Yes	Yes	No	Yes	−0.08	No	Yes	0.74
16c	−5.64	1.46	90.90	0.26	No	Yes	Yes	Yes	Yes	No	Yes	−0.09	No	Yes	0.76
17a	−5.44	1.62	90.00	0.28	No	Yes	Yes	Yes	Yes	No	Yes	−0.15	No	Yes	0.73
18a	−5.51	1.24	90.72	0.29	No	Yes	Yes	Yes	Yes	No	Yes	−0.085	No	Yes	0.80
19a	−4.21	1.74	96.38	0.28	No	Yes	Yes	Yes	No	No	No	0.77	No	No	1.18
20a	−4.63	1.50	97.61	0.11	No	Yes	Yes	Yes	Yes	No	No	0.75	No	No	1.23
20b	−5.28	1.51	95.95	0.10	No	Yes	Yes	Yes	Yes	No	No	−0.04	No	No	1.19

**Table S4:** Binding affinity of the studied compounds

No	pIC50	Binding affinity (Kcal/mol)	No	pIC50	Binding affinity (Kcal/mol)
8b	6.5968	−9.4	11g	6.2048	−9.4
9a	6.1007	−9.5	12b	5.5497	−8.5
9b	5.6740	−9.5	15a	5.2232	−7.9
10a	6.3269	−9.2	15b	5.2831	−8.1
10b	7.7958	−9.9	16a	5.4723	−8.5
10c	6.8386	−9.6	16b	5.3106	−8.3
10d	6.70114	−9.3	16c	6.5482	−9.4
10e	7.2518	−9.7	17a	6.2365	−9.1
10f	7.1611	−9.6	18a	6.6516	−9.2
10g	6.3777	−9.3	19a	5.2306	−8.3
10h	5.8551	−9.3	20a	5.5867	−8.7
11a	6.7399	−9.4	20b	6.0282	−9.2
11b	7.2441	−9.5			

## Figures and Tables

**Figure 1 f1-turkjchem-46-3-687:**
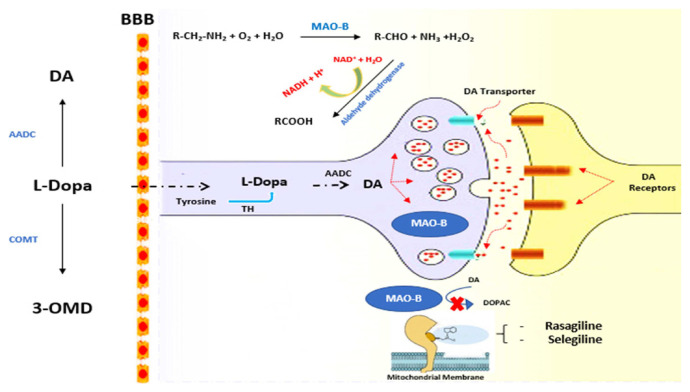
Mechanism of interaction of monoamine oxidase-B (MAO-B) inhibitors. AD = dopamine, L-Dopa = levodopa, AADC = aromatic L-amino acid decarboxylase, 3-OMD = 3-O-methyldopa, COMT = catechol-O-methyltransferase, TH = tyrosine hydroxylase, and DOPAC = 3, 4-dihydroxyphenylacetic acid.

**Table 1:** Chemical structures of unsaturated ketone derivatives and their corresponding experimental activities.

No	Structure	pIC50	No	Structure	pIC50
8b		6.5969	12a		5.0762
9a		6.1007	12b		5.5498
10a		6.3270	15a		5.2233
10b		7.7959	15b		5.2832
10c		6.8386	16a		5.4724
10d	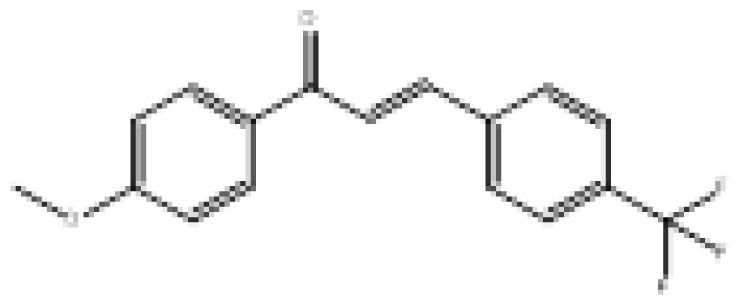	6.7011	16b	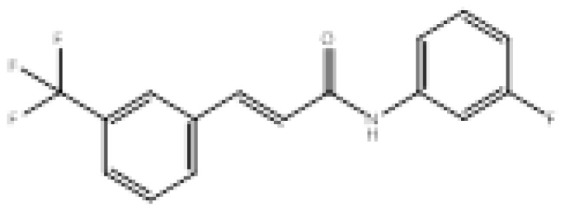	5.3107
10e	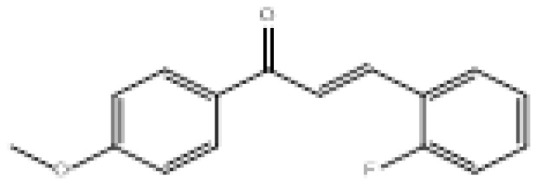	7.2518	16c	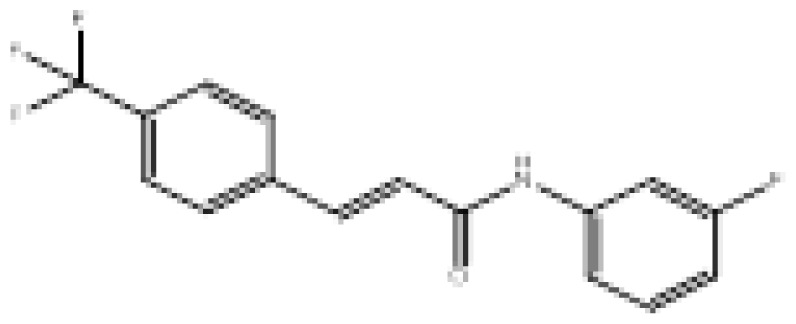	6.5482
10f	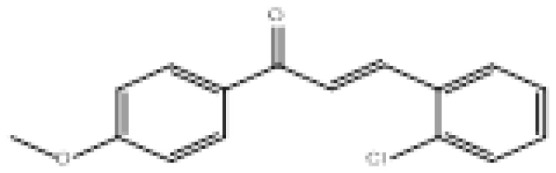	7.1612	17a	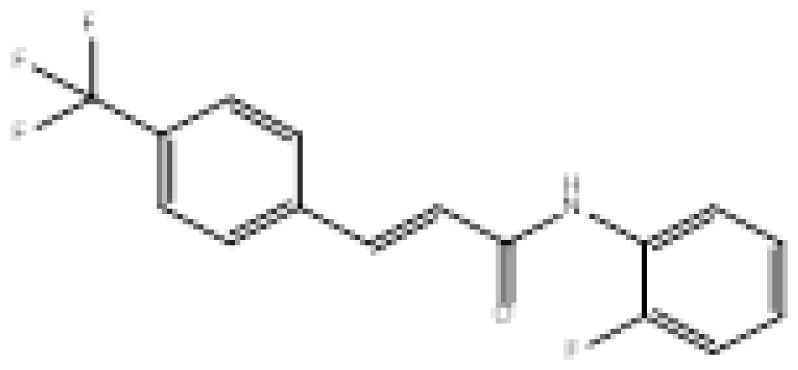	6.2366
10g	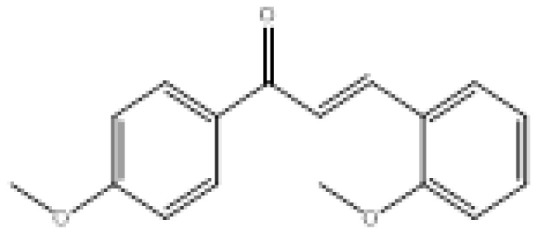	6.3778	18a	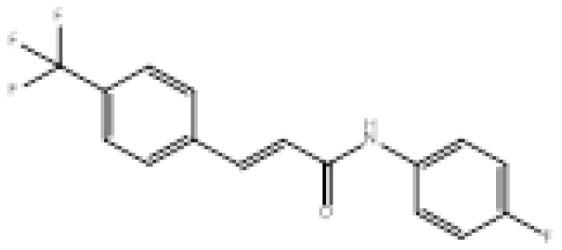	6.6517
10h	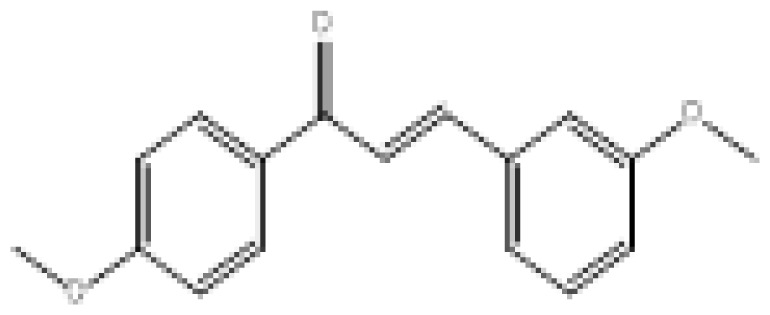	5.8551	19a	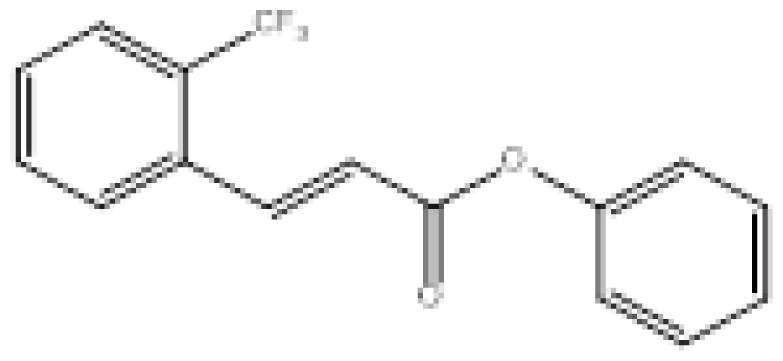	5.2306
11a	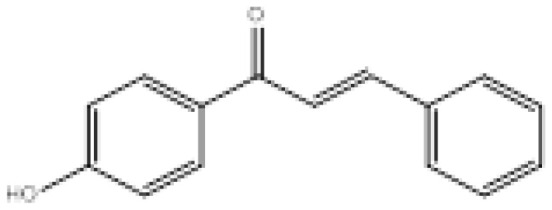	6.7399	20a	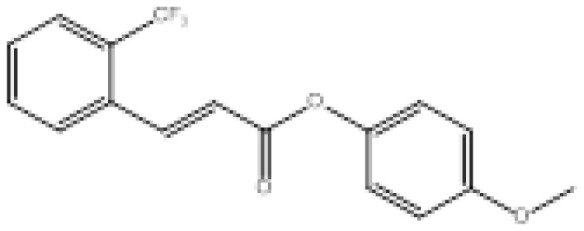	5.5867
11b	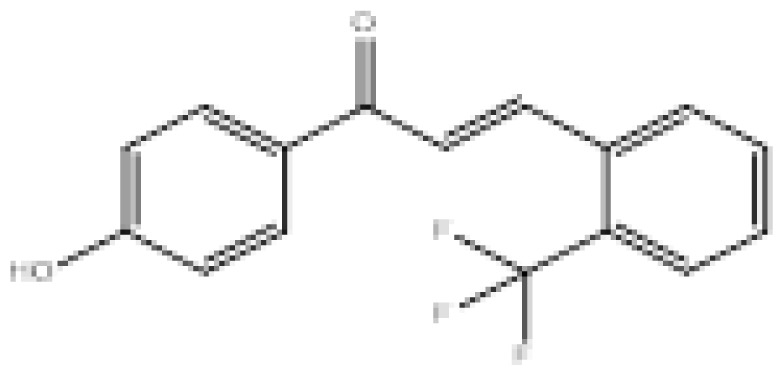	7.2441	20b	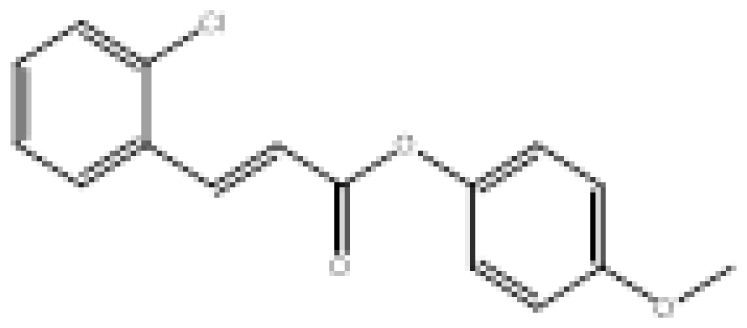	6.0283
11g	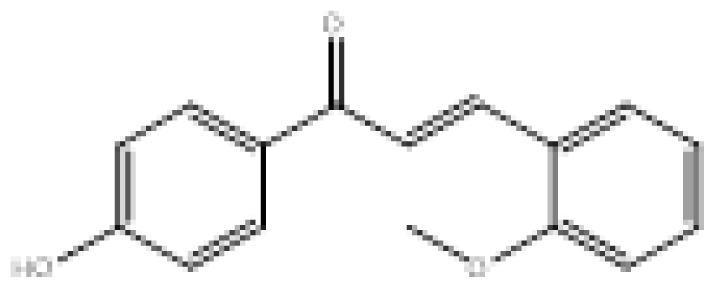	6.2048			

T indicates test set compounds.

**Table 2:** VIF and p-values of the selected descriptors.

Statistique	BCUT_SMR_2	logP(o/w)	SlogP_VSA4	Vsurf_IW3
Tolérance	0.5117	0.6216	0.5946	0.6546
VIF	1.9545	1.6087	1.6817	1.5277
p-value	0.0012	0.0092	<0.0001	0.0007
R_square	−0.34	0.44	0.72	−0.31

**Table 3:** Correlation matrix of the selected descriptors.

Variables	BCUT_SMR_2	logP(o/w)	SlogP_VSA4	vsurf_IW3	pIC50
BCUT_SMR_2	1.0000				
logP(o/w)	−0.4640	1.0000			
SlogP_VSA4	−0.5413	0.0986	1.0000		
vsurf_IW3	0.2441	−0.5042	0.1923	1.0000	
pIC50	−0.3386	0.4451	0.7195	−0.3148	1.0000

**Table 4:** Values of selected descriptors, experimental activity, and predicted activity.

No	Molecular descriptors				pIC50_Obs	2D-QSAR	
	logP(o/w)	SlogP_VSA4	vsurf_IW3	BCUT_SMR_2		pIC50_Pred	pIC50_Loo
8b	4.9518	33.4189	2.4626	0.7751	6.5968	6.7780	6.8746
9a	4.0580	30.2334	2.8303	0.8189	6.1007	6.2360	6.2645
10a	4.0210	30.2334	2.1943	0.8309	6,3269	6.5958	6.6497
10b	4.9538	33.4189	0.4323	0.7648	7.7958	7.5893	7.4689
10c	4.9928	33.4189	2.0546	0.7876	6.8386	7.0902	7.1913
10e	4.1720	30.2334	1.8544	0.8309	7.2518	6.8420	6.7629
10f	4.6110	30.2334	2.3715	0.8311	7.1611	6.8939	6.8292
10g	3.9750	30.2334	1.8080	0.7969	6.3777	6.4492	6.4735
11g	3.7110	30.2334	2.8445	0.8335	6.2048	6.1328	6.1054
12b	4.0930	3.1856	1.5152	0.8826	5.5497	5.8354	5.8976
15a	4.1648	6.3712	1.8161	0.7838	5.2232	5.0928	5.0162
15b	3.8220	3.1856	3.0879	0.9392	5.2831	5.4470	5.5726
16a	4.6258	6.3712	1.5711	0.7690	5.4723	5.3691	5.3122
16b	4.6648	6.3712	1.2923	0.8091	5.3106	5.8565	5.9500
16c	4.6278	6.3712	0.7767	0.8543	6.5482	6.4438	6.4168
17a	4.5888	6.3712	1.2236	0.8543	6.2365	6.2213	6.2190
18a	4.5908	6.3712	0.2897	0.8543	6.6516	6.6357	6.6276
19a	4.1450	3.1856	2.0563	0.8543	5.2306	5.3892	5.4115
20a	4.1010	3.1856	2.5898	0.8543	5.5867	5.1253	5.0201
20b	4.6910	3.1856	2.3541	0.8718	6.0282	5.7528	5.6333
10d*	4.9558	33.4189	1.4270	0.8270	6.7014	7.6778	-
10h*	4.0140	30.2334	2.1155	0.7972	5.8551	6.3405	-
11a*	3.7570	30.2334	2.9518	0.8543	6.7399	6.2909	-
11b*	4.6898	33.4189	1.6233	0.8175	7.2441	7.3417	-
12a*	4.4358	6.3712	1.5940	0.7690	5.0762	5.2381	-

* Represents compounds of test set, pIC50_Pred represents the value of activity predicted by the created model and pIC50_LOO represents the value of activity predicted by leave-one-out cross-validation method.

**Table 5:** Accepted 2D-QSAR model validation tools.

Parameters	Interpretation	Acceptable value	Generated 2D-QSAR model
r^2^	Coefficient of determination	≥0.6	0.88
r^2^_adjusted_	Adjusted R-squared	>0.6	0.84
Q^2^_LOO_	Coefficient of determination for internal validation	>0.5	0.81
R^2^_m cv_	R2m cross validation parameter	>0.5	0.57
cR_p_^2^	Coefficient of determination for *Y*-randomization	>0.5	0.81
R^2^_test_	Coefficient of determination of external validation	>0.6	0.71
Q^2^ (F)	The regression slope passing through the origin	>0.5	0.52
K	The regression slope passing through the origin (plot of experimental versus predicted activities)	0.85 ≤ k ≤ 1.15	0.96
K′	The regression slope passing through the origin (plot of predicted versus experimental activities)	0.85 ≤ k′ ≤ 1.15	1.04
