# Supplementary figures and images for: Computational investigation of unsaturated ketone derivatives as MAO-B inhibitors by using QSAR, ADME/Tox, molecular docking, and molecular dynamics simulations
Source: Turk J Chem. 2021 Dec 18;46(3):687–703. doi: 10.55730/1300-0527.3360 (PMC10503977; doi:10.55730/1300-0527.3360)

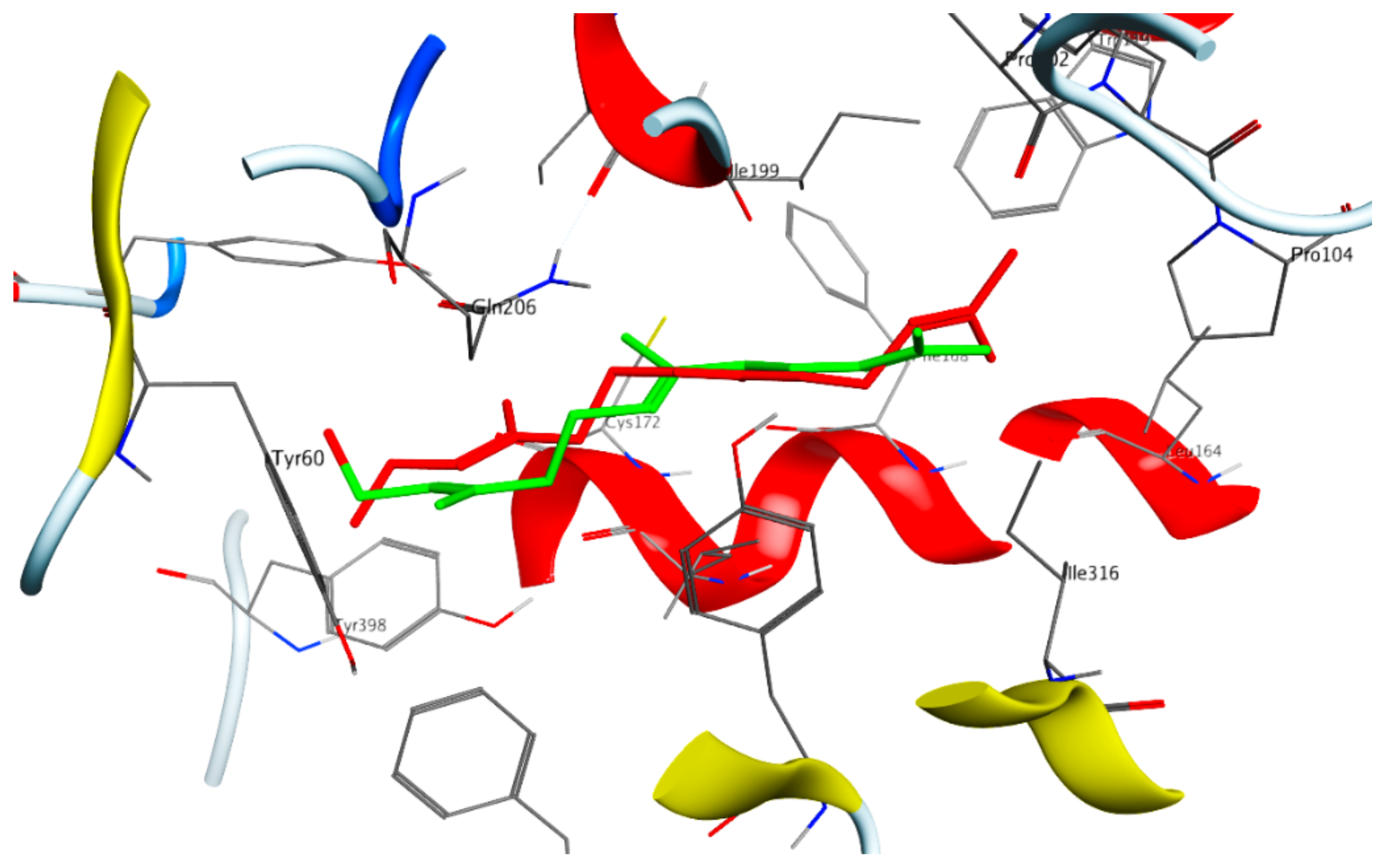

Supplement: Figure S1 — The superposition between the native (green color) and the redocked (red color) ligands into the binding site of MAO-B enzyme. [file turkjchem-46-3-687s1.tif]
